# Differential Effects of Cannabinoid Receptor 2 Agonists on HIV Replication and Inflammatory Activation in Monocyte-Derived Macrophages and Induced Pluripotent Stem Cell-Derived Microglia

**DOI:** 10.1007/s11481-025-10254-x

**Published:** 2025-10-11

**Authors:** Alexander Starr, Sara Rathore, Marzieh Daniali, Peter J. Gaskill, Cagla Akay-Espinoza, Kelly L. Jordan-Sciutto

**Affiliations:** 1 Department of Oral Medicine, School of Dental Medicine, University of Pennsylvania, 240 S. 40th St, Rm 312 Levy, Philadelphia, PA 19104 USA; 2 Department of Pharmacology & Physiology, Drexel University College of Medicine, Philadelphia, PA 19102 USA

**Keywords:** Cannabinoid receptor 2, Human immunodeficiency virus, Inflammation, JWH-133, Myeloid lineage, Microglia

## Abstract

Emerging evidence suggests brain-resident myeloid cells, including perivascular macrophages and microglia, provide a reservoir for HIV infection in the central nervous system (CNS), and their inflammatory activation is a proposed pathogenic mechanism in HIV-associated neurocognitive disorders (HAND). We investigated whether cannabinoid receptor 2 (CB_2_), an immunomodulatory receptor expressed in myeloid cells, regulates viral replication and inflammation in HIV-infected macrophages and microglia. Using the synthetic CB_2_-specific agonist JWH-133, we found that CB_2_ activation reduced HIV replication in primary human monocyte-derived macrophages (MDMs) and human induced pluripotent stem cell-derived microglia (iMg) at differing doses, corresponding to the basal expression of *CNR2*, which encodes CB_2_, and related endocannabinoid transcripts in each cell type. JWH-133 broadly reduced release of cytokines from HIV-infected MDMs but not iMg. RNA-seq revealed that CB_2_ agonism primarily altered interferon and integrated stress response pathways in MDMs while altering homeostatic pathways, including synapse maintenance and phagocytosis, in iMg. Further analyses in iMg revealed that NLRP3 inflammasome activation, but not priming, was reduced by CB_2_ activation, which did not inhibit HIV-induced nuclear factor kB activation. This study identifies key differences in CB_2_ response between myeloid lineage cell types and implicates CB_2_-specific agonists as promising candidates for the regulation of HIV-associated neuroinflammation.

**Graphical Abstract:**

Created in BioRender. Espinoza, C. (2025) https://BioRender.com/mxfla3i

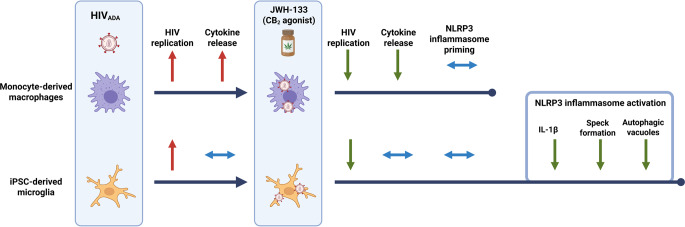

**Supplementary Information:**

The online version contains supplementary material available at 10.1007/s11481-025-10254-x.

## Introduction

Human immunodeficiency virus (HIV) remains a significant public health concern, with almost 40 million people living with HIV (PWH) globally (https://aidsinfo.unaids.org/). While the success of anti-retroviral therapy (ART) has increased the length and quality of life for PWH, longer chronic infection has also resulted in an increase in comorbidities (Gallant et al., 2017), including a range of neurological complications that have been reported to impact up to 50% of PWH according to some estimates (Saylor et al., 2016), which may result from persistent HIV infection in the CNS, as well as the inflammatory response to infection within this compartment (Ellis et al. 2023; Tavasoli et al., 2023; Williams & Naudé, 2024).

HIV enters the central nervous system (CNS), via direct and indirect means, early after infection and establishes infection in CD4^+^ cells, predominantly brain-resident myeloid lineage cells, including perivascular macrophages and parenchymal microglia (Ghorpade et al., 1998; Spudich et al., 2019; Thompson et al., 2011). Recent reports demonstrate that myeloid lineage cells constitute viral reservoirs even in individuals on suppressive ART (Cochrane et al. 2022; Gabuzda et al., 2023; Kreider & Bar, 2022; Tang et al., 2023; Thompson et al., 2011; Veenhuis et al., 2023; Wallet et al., 2019). HIV-associated neurocognitive disorders (HAND) – an umbrella term for mood, memory, cognitive, and motor dysfunction that can develop in PWH – also persists despite widespread ART use (Gelman, 2015; Heaton et al., 2011; Kolson, 2022; Sakamoto et al., 2013; Saylor et al., 2016). Therefore, investigating approaches to mitigate viral reservoirs in myeloid lineage cells and the downstream consequences is important even in the setting of successful virologic control.

In myeloid lineage cells, one common consequence of HIV infection is immunologic activation, which encompasses changes in gene and protein expression and cytokine/chemokine production, all aimed to counter pathogens and recruit additional immune cells (Ghorpade et al., 2005; Sheng et al., 2000; Yoshioka et al., 1995; Younas et al., 2016). In brain-resident myeloid lineage cells, sustained immune activation induces the production and secretion of cytokines, such as interleukin (IL)−6 and tumor necrosis factor (TNF)-α, which are elevated in the cerebrospinal fluid of PWH with HAND and exhibit varying degrees of correlation with disease risk (Anderson et al., 2021; de Almeida et al. 2016; Letendre et al., 2011; McGuire et al., 2015; Meeker et al., 2011; Nightingale et al., 2016; Nolting et al., 2012; Yuan et al., 2015; Yuan et al., 2013). Studies show that regulating myeloid cell-mediated inflammation is neuroprotective in several in vitro models of HIV-induced CNS injury (Ambrosius et al., 2019; Cross et al. 2011; Kolson, 2022). Importantly, although macrophages and microglia are functionally similar, some differences exist, including ontogeny, tissue distribution, homeostatic and immunologic responsibilities, and potentially the magnitude of response to specific insults (Bennet & Bennet 2020; Chen et al. 2023; Eggen et al 2019; Gosselin et al., 2017; Silvin et al., 2022; Silvin et al., 2022; Umekawa et al., 2015). Thus, identification and mitigation of HIV-associated inflammation should consider cell type-specific inflammation and response to potential therapeutic approaches.

While previous clinical trials aimed at mitigating HIV-associated inflammation using various anti-inflammatory strategies have failed, evidence supports inflammation as a persistent feature in PWH (Eden et al., 2016; Rubin et al., 2018; Vera et al., 2016). In attempts to mitigate HIV-associated inflammation, one attractive immunoregulatory target is the endocannabinoid system, which counters the release of inflammatory cytokines via the action of the endogenous ligand 2-arachidonoylglycerol on cannabinoid receptor 2 (CB_2_), a G_i_/G_o_ G protein-coupled receptor (Basavarajappa et al., 2017; Benito et al., 2008; Howlett & Abood, 2017; Rom & Persidsky, 2013; Soethoudt et al., 2017). Cannabinoids, including endocannabinoids, phytocannabinoids delta-9-tetrahydrocannabinol (Δ9-THC) and cannabidiol (CBD), as well as synthetic cannabinoids can act on CB_2_ and the neuromodulator CB_1_ to elicit psychoactive effects in the absence of a specific pathology (Gilbert et al., 2007; Kaplan, 2013; Kelley & Thayer, 2004; Kozela et al., 2010, 2017; Navarro et al., 2018; Roloff & Thayer, 2009; Wu & Thayer, 2020; Xu et al., 2017). Several reports have shown that cannabinoids attenuate HIV infection and/or replication in T-cells, macrophages, dendritic cells and ex vivo human fetal microglia cultures (Agudelo et al., 2015; Costantino et al. 2012; Ramirez et al., 2013; Rock et al., 2007; Williams et al., 2014). Efforts to separate the anti-inflammatory effects from the psychotropic effects have revealed differential activities of CB_1_ and CB_2_, which exhibit differential tissue expression and agonism with endocannabinoids and phytocannabinoids. As suggested by their function, CB_1_ is primarily expressed on neurons, while CB_2_ is expressed in immune cells, including myeloid cells, in both the CNS and periphery (Bossong and Niesink 2010; Turcotte et al., 2016). Considering the proximity of CNS-resident myeloid lineage cells to neurons, careful selection of pharmacologic agents is required to achieve immunomodulation without impacting neurotransmission. Several studies indicate that the anti-inflammatory properties of CB_2_ agonism might include the modulation of the NLR family pyrin domain-containing 3 (NLRP3) inflammasome, largely in immunomodulatory cells (Han et al. 2018, 2019; Ke et al., 2016; Shao et al., 2014; Sheng et al., 2019; Yu et al., 2019).

In this study, we investigated whether previous observations showing that CB_2_-specific agonists suppressed HIV replication in monocyte-derived macrophages (MDMs) extended to human induced pluripotent stem cell (iPSC)-derived microglia (iMg) (Ramirez et al., 2013; Rock et al., 2007). Using equivalent input of HIV-1 inoculum and the CB_2_-specific agonist JWH-133 along with the CB_2_-specific antagonist/inverse agonist SR-144,528, we demonstrate that CB_2_ agonism exerts a cell type-specific, nuanced impact on viral replication, release of proinflammatory cytokines, and gene expression. We also identify the NLRP3 inflammasome, which is induced by HIV infection and mediates IL-1β release and pyroptosis, as an immunomodulatory target of CB_2_-signaling in iMg.

## Materials and Methods

### Antibodies and Primers

The following primary antibodies were used at indicated concentrations: FITC-tagged anti-CD4 (1:250; cat. no: 130-114-531; Miltenyi Biotec, Waltham, MA), PE-tagged anti-CCR5 (1:250; cat no: 130-117-356; Miltenyi Biotec), mouse anti-HIV p24 Gag (1:200; cat. no: HRP-20068; NIH HIV Reagent Program), rabbit anti-NLRP3 (1:250; cat. no. ab263899; Abcam, Waltham, MA), mouse anti-ASC (1:400; cat. no: sc-271054; Santa Cruz Biotechnology, Dallas, TX), and rabbit anti-NF-kB p65 (1:10200; cat no: 8242; Cell Signaling Technologies, Danvers, MA). FITC- and Cy3-conjugated secondary goat anti-mouse and anti-rabbit antibodies (all from Jackson ImmunoResearch, West Grove, PA) were used at 1:200.

Custom TaqMan assay plates preloaded with the following human primer/probe pairs (Thermo Fisher Scientific, Waltham, MA) were used: *GAPDH*, Hs02786624_g1; *CD4*, Hs01058407_m1, *CCR5*, Hs99999149_s1; *CXCR4*, Hs00607978_s1; *CNR1*, Hs01038522_s1; *CNR2*, Hs00361490_m1; *GPR55*, Hs00271662_s1; *IL1B*, Hs01555410_m1; *IL18*, Hs01038788_m1; *NLRP3*, Hs00918082_m1; *PYCARD*, Hs01547324_gH; *IL6*, Hs00174131_m1; *TNFA*, Hs00174128_m1; and *CASP1*, Hs00354836_m1.

### MDM and iMg Differentiation

MDMs were differentiated as previously described (Cross et al. 2011). Briefly, primary human monocytes of healthy volunteers, which were isolated using the EasySep Human Monocyte Enrichment kit (STEMCELL Technologies), were obtained from the University of Pennsylvania Human Immunology Core. The cells were plated at a concentration of 2.4 × 10^5^ cells/mL and differentiated in Dulbecco’s modified Eagle’s medium supplemented with 10% human serum and 20 ng/mL macrophage colony-stimulating factor for seven days. For iMg, common myeloid progenitors generated from iPSCs of adult human fibroblasts were purchased from the CHOP Stem Cell Core and differentiated to iMg over 11 days in RPMI 1640 (HyClone) containing 10% fetal bovine serum, 20 ng/mL macrophage colony-stimulating factor, 100 ng/mL IL-34, and 50 ng/mL transforming growth factor beta, as previously described (Ryan et al., 2020).

### HIV Infection and Cannabinoid Treatments

Fully differentiated MDMs and iMg were treated with HIV_ADA_, a lab-adapted HIV-1 strain that utilizes the CCR5 coreceptor for entry and thus termed macrophage-tropic, at a concentration of 1 ng/mL HIV p24 Gag on day post-infection (DPI) 0. After 24 h (DPI 1), HIV-containing medium was removed and indicated treatments were added in fresh media. JWH-133 (cat. no. 1418; Axon Medchem, Reston, VA) and SR-144,528 (cat. no. 1924; Axon) were reconstituted in dimethyl sulfoxide (DMSO) and serially diluted to ensure a uniform final volume of vehicle across treatment groups. To evaluate receptor specificity, SR-144,528 was added for 1 h prior to JWH-133 treatment to ensure receptor saturation. In MDM, every 48 h until DPI 9, 100% of culture medium was replaced with fresh medium and cannabinoid treatments were added after each medium replacement. In iMg, the same procedure was followed, but only 80% of culture medium was replaced.

Culture supernatants were collected at each medium replacement. For lipopolysaccharide (LPS) + ATP treatments, which were used as the positive control of inflammasome activity, uninfected cultures were treated with 1 ng/ml LPS from *Escherichia coli* O55:B5 (Sigma-Aldrich) or 23.5 h beginning on DPI 8, with additional treatment with 2.5 mM ATP for 30 min prior to supernatant collection.

###  Measurement of p24 Gag and Cytokine Levels

Alphalisa kits (all from Revvity, Waltham, MA) were used to measure HIV p24 Gag (cat. no. AL291C), TNF-α (cat. no. AL3157C), IL-6 (cat. no. AL223C), and IL-1β (cat. no. AL220C) in duplicate culture supernatant samples of MDMs and iMg (minimum three pooled wells/treatment) according to the manufacturer’s protocols. MDM supernatants were diluted at 1:20 for the HIV p24 Gag assay. For unbiased cytokine assays, undiluted supernatants were evaluated in duplicate using the Bio-Plex Pro Human Cytokine Screening Panel, 48-Plex (cat. no. 12007283; Bio-Rad, Hercules, CA).

### Immunofluorescence Staining

iMg in glass-like polymer-coated culture plates (Cellvis, Mountain View, CA) were fixed with 4% paraformaldehyde for 10 min, permeabilized with 0.1% Triton-X in phosphate-buffered saline, and blocked with 5% normal goat serum and 0.5% bovine serum albumin in phosphate-buffered saline. For staining with unconjugated antibodies, the cultures were incubated with primary antibodies diluted in blocking solution overnight at 4 °C, followed by incubation with secondary antibodies and 4´,6-diamidino-2-phenylindole, dihydrochloride (DAPI, 1:2500) at 25 °C for 30 min. Staining with preconjugated primary antibodies to CD4 and CCR5 were performed with incubation with antibodies for 1 h at room temperature. Stained cells were stored at 4 °C in PBS until image capture, for a maximum of 24 h.

### Imaging and Quantification

Images were captured using a Keyence BZ-X710 fluorescent microscope (Keyence, Itasca, IL) affixed with a 40-× objective and DAPI, FITC, and Cy3 filter cubes. The automatic stage was used to capture 3 × 3 grids of images around a set center point in each well. Grids were stitched and merged using the BZ-X analyzer software (Keyence). The same settings for fluorescence intensity and size were used to define DAPI^+^ nuclei and positive staining for CD4, CCR5, NLRP3, and ASC. NLRP3^+^/ASC^+^ specks were defined as double-positive puncta that were ≥ 0.3 μm in size. These settings were then used to uniformly evaluate counts/intensities in all images in experiments.

### RNA Isolation and Sequencing

Bulk RNA sequencing was performed by Azenta Life Sciences/Genewiz (Burlington, MA). Briefly, following supernatant collection on DPI 9, MDMs and iMg were lysed in wells using RLT buffer (RNeasy Mini Kit; Qiagen, Germantown, MD), and pooled RNA was isolated using the manufacturer’s protocol. RNA purity and concentration were confirmed using Nanodrop (Thermo Fisher Scientific), and RNA Clean & Concentrator (Zymo, Irvine, CA) was used for samples that did not pass the initial quality control, defined using the following cutoffs: 260/280 absorbance ratio of > 2, 260/230 absorbance ratio of > 1.8, and minimum concentration of > 50 ng/µL. After quality confirmation of the purified RNA, samples were submitted to Azenta Life Sciences/Genewiz for mRNA-enriched library preparation and sequencing via Illumina HiSeq, with an average read depth of approximately 22 million reads/sample. Reads were trimmed using Trimmomatic v.0.36 and mapped to the Homo sapiens GRCh38 reference genome using STAR aligner v.2.5.2b. Raw gene counts were calculated using featureCounts from the Subread package v.1.5.2. These raw counts were read into NOISeq v.2.44.0, low counts were filtered out by minimum 5 counts/million reads and normalized by reads/kilobase per million mapped reads (Tarazona et al., 2015). Significantly differentially expressed genes (DEGs) were defined by a cutoff of q = 0.9. Cross-cell type analyses used DESeq2. DEGs were ranked by the probability of differential expression, and top 30 DEGs were evaluated using hierarchical clustering of normalized counts with Heatmapper (Babicki et al., 2016). All DEGs were subjected to gene enrichment analysis using ShinyGO 0.77, and top 10 enriched gene ontology biologic process/KEGG pathways were identified (Ge et al., 2020; Kanehisa et al., 2021). All sequencing data are available in the NCBI GEO database (https://www.ncbi.nlm.nih.gov/geo/) with the accession number GSE250616.

### Reverse Transcription and Quantitative Polymerase Chain Reaction

RNA was isolated as described above, and 250 ng RNA were reverse transcribed using the RT^2^ First Strand kit (cat. no. 330404; Qiagen), and cDNA was loaded onto preconfigured custom TaqMan array plates with TaqMan Fast Advanced Master Mix (cat. no. 4444556; Applied Biosystems, Foster City, CA). Quantitative reverse transcription (qRT)-polymerase chain reaction (PCR) was performed using the QuantStudio 3 Real Time PCR system (Applied Biosystems). Results were reported as ΔΔCt-determined log_2_ fold changes, normalized to *GAPDH* levels.

### NF-kB Translocation Assay

Following immunofluorescent staining for cell nuclei (DAPI, Sigma-Aldritch), cell bodies (Cell Mask Deep Red, ThermoFisher Scientific) and NF-κBp65 (Cell signaling), iMg seeded in 96-well plates were imaged on a Cell Insight CX7 High Content screening platform (CX7; ThermoFisher Scientific). Four wells were imaged for each condition, acquiring approximately 1000–2000 cells/well (4000–8000 cells/condition). Images were acquired with a fixed exposure time and intra-well autofocusing with every field using parameters available in Table 1. Images were analyzed using HCS studio software and the Cellomics Colocalization bio-application (Cellomics, ThermoFisher Scientific). This analysis creates a binary mask for the cytoplasmic (Cell Mask Deep Red) and nuclear (DAPI) regions of interest (ROI) and quantifies the average intensity of NF-κB staining in each ROI for every cell. The average intensity of NF-κB in the nuclear ROI was divided by the average intensity of NF-κB in the cytoplasmic ROI (NF-κB _nuclear_/NF-κB _cytoplasmic_). This was calculated for in each individual cell, generating a nuclear colocalization ratio for each individual cell. This analysis quantifies the relative amount of NF-κB in the nucleus, while also controlling for differences in cell size and total NF-κB amount among different cells. The nuclear colocalization ratio for all cells from a particular condition (3 wells per condition) was then averaged, and the average ratios from each condition were pooled across all donors to generate the overall average for the N of 6. This overall average was then used for statistical comparisons.

**Table 1:** Parameters used for the high content analysis of NF-κB colocalization assay

Condition	Value
Ch1: SmoothFactor	2
Ch1: Thresholding (Fixed)	150
BackgroundCorrectionCh1	2
Object.Ch1.Average Intensity.Ch1	0-65535
Ch2: SmoothFactor	1
Ch2: Thresholding (Fixed)	220
BackgroundCorrectionCh2	2
Ch2: Segmentation (Intensity)	−1750
ObjectAreaCh2	170.32–1155.189
Object.Average Intensity.Ch2	0–65,000
Ch3: SmoothFactor	0
Ch3: Thresholding (Fixed)	400
BackgroundCorrectionCh3	2
Ch3: Segmentation (Intensity)	−1850
ObjectAreaCh3	193.144–16199197.97
Object.Average Intensity.Ch3	65,535
ROI.A.Mask Ch	Channel 1 (DAPI)
ROI.A.Target_I	Channel 2 (NF-kB)
ROI.B.Mask Ch	Channel 3 (CMDR)
ROI.B.Target_I	Channel 2 (NF-kB)
ROI.B.Exclude	Channel 1 (DAPI)
RejectBorderObjects	Y

### Toxicity, Caspase Activity, and Autophagy Assays

Cytotoxic potential of HIV_ADA_ and CB_2_-specific cannabinoids was assayed using the Cytotoxicity Detection^PLUS^ kit (cat. no. 04744926001; Roche, Indianapolis, IN), according to the manufacturer’s protocol, with Triton-X-lysed cells used as positive control for maximal toxicity. Caspase 1 activity was measured in duplicate with the Caspase-Glo 1^®^ inflammasome assay (cat. no. G9951; Promega, Madison, WI), according to the manufacturer’s protocol. Autophagosomes in cultured cells were quantified in triplicate using the Autophagy Assay kit (cat. no. ab139484; Abcam) with a luminescence plate reader (Luminoskan Ascent, Thermo Fisher Scientific), according to the manufacturer’s protocol. Treatment with rapamycin and chloroquine for 18 h served as positive controls.

### Statistical Analysis

Initial HIV replication experiments in MDMs were performed using cells from seven donors, with follow-up supernatant analysis in 5–6 additional donors. RNA analyses included three MDM donors and three distinct iMg lines. Supernatants from three iMg lines were used for ELISA. Immunofluorescence staining was performed in two iMg lines, with data averaged and represented by three differentiations per iMg line to ensure that the observed molecular changes were consistent. Analysis of NF-kB was performed in three distinct iMg lines in two separate experiments, using a distinct differentiation of each line each experiment. Cytokine assay results were evaluated using Student’s *t* test to solely compare HIV + Veh with HIV + JWH in all samples. Longitudinal analyses were evaluated by two-way analysis of variance, and the remaining analyses were evaluated by one-way analysis of variance.

## Results

### CB_2_-Specific Agonists Dose-Dependently Impair HIV Infection in MDMs and iMg

**Fig. 1:**
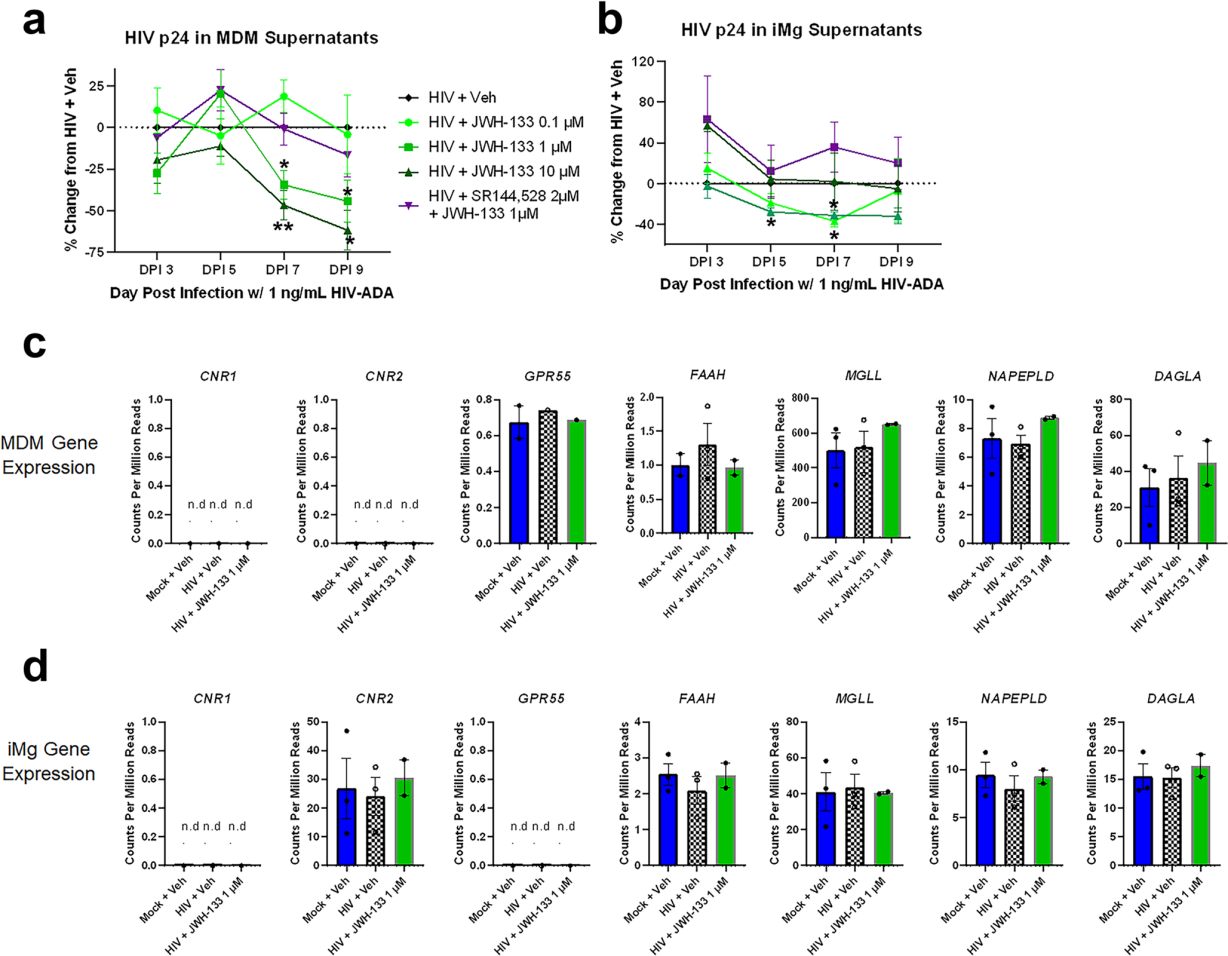
CB_2_ agonists dose-dependently reduce HIV replication in macrophages and microglia.** a**,** b.** HIV p24 levels in supernatants of monocyte-derived macrophages (MDMs, *n* = 7) (**a**) and human induced pluripotent stem cell-derived microglia (iMg, *n* = 3) (**b**), normalized to the HIV + vehicle. Two-way ANOVA with Dunnett’s correction for multiple comparisons. * *p* < 0.05, ** *p* < 0.01. **c**,** d.** Bulk RNA-seq data showing count/million reads of key endocannabinoid system genes in MDMs (**c**) and iMg (**d**)

**Fig. 2:**
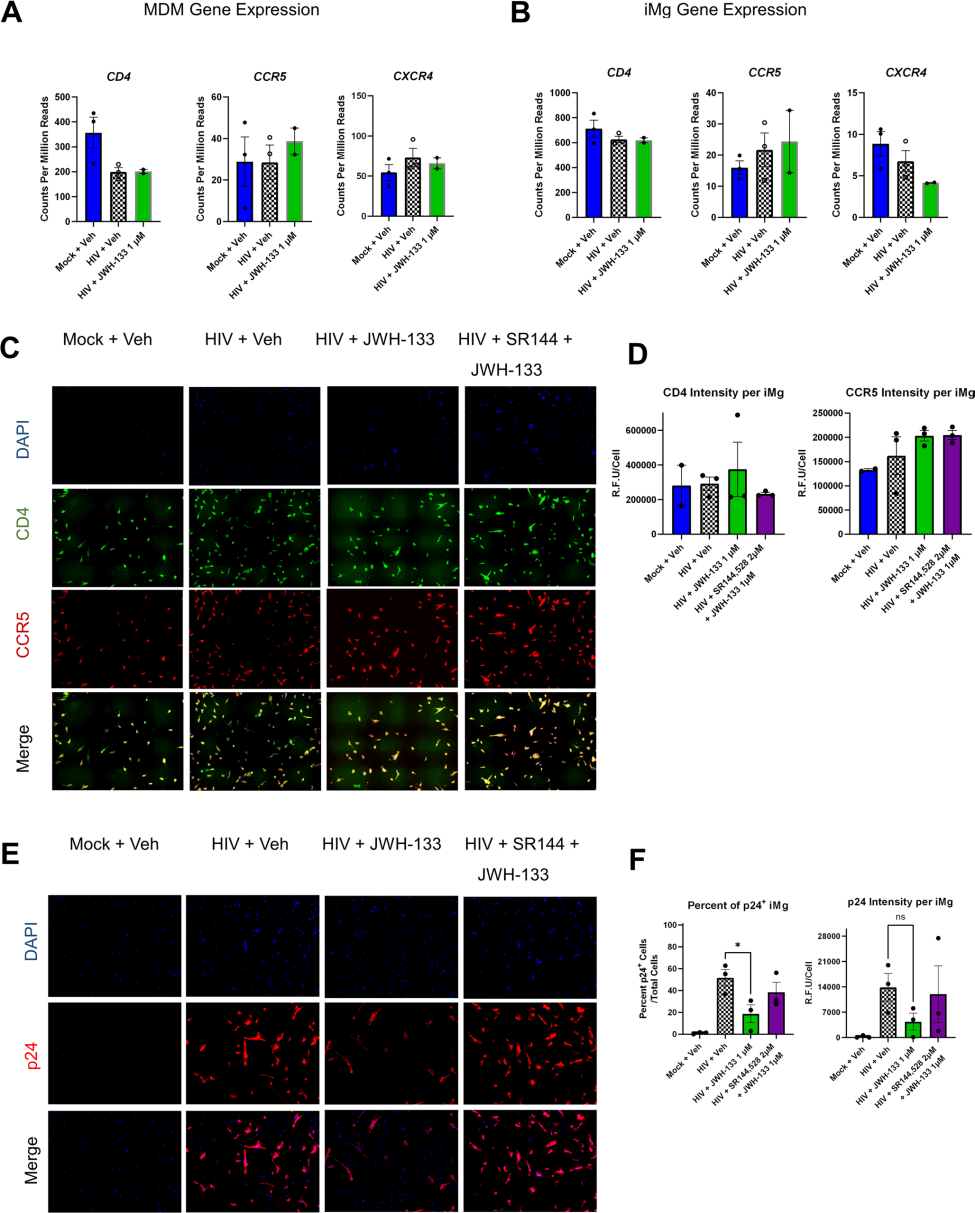
CB_2_ activation reduces the number of infected cells without altering HIV receptor expression.** a**,** b.** Bulk RNA-seq analysis showing the expression of key endocannabinoid system genes in (**a**) MDMs and (**b**) iMg. **c.** Representative images of iMg immunostained for CD4 (red), CCR5 (green), and DAPI (blue). **d.** Quantification of CD4 and CCR5 expression. Each symbol represents the average of 2 wells/condition. *n* = 3 differentiations from 2 iMg lines. **e.** Representative images of iMg immunostained for HIV p24. **f.** Quantification of CD4 and CCR5 expression. Each symbol represents the average of 2 wells/condition. *n* = 3 differentiations from 2 iMg lines. One-way analysis of variance (ANOVA) with Dunnett’s correction for multiple comparisons. * *p* < 0.05; ns, not significant

HIV replication on DPI 9, measured by HIV p24 Gag protein concentrations in supernatants, was approximately 40× greater in MDMs than in iMg exposed to the same inoculum of HIV_ADA_ (1 ng/mL p24 Gag) (Fig. 1a and b; Supplementary Fig. S1a, S1-b), although *CD4* and *CCR5* mRNA levels at baseline were comparable between MDMs and iMg (Fig. 2a and b; Supplementary Fig. S3a). This could be, in part, due to differences in replication kinetics: MDMs from some donors reached near-maximal p24 Gag levels by DPI 7 or DPI 9, at which time p24 Gag production had not still reached a plateau in iMg (Supplementary Fig S1a, S1b). Due to the inherent variation in susceptibility to HIV infection among human donors, each MDM batch was normalized to its own HIV + Veh for downstream analyses. Importantly, cell death was not observed at DPI 9 in MDMs or iMg in any treatment group, indicating levels of p24 Gag and other supernatant components were likely secreted, as opposed to being released from damaged cells (Supplementary Fig. S2).

Treatment with JWH-133 significantly reduced HIV p24 Gag in both MDMs and iMg; however, the effective dose and timing differed between the two cell types. MDMs exhibited a classic dose response; 0.1 µM JWH-133 showed variable effect, 1 µM JWH-133 reduced p24 Gag levels by approximately 30% on DPI 7, and 10 µM JWH-133 further reduced p24 Gag levels by 20% (Fig. 1a). In MDMs, SR-144,528 pretreatment abrogated the effect achieved with 1 µM but not 10 µM JWH-133. Conversely, in iMg, JWH-133 treatment led to a significant reduction in p24 Gag levels by DPI 5 at doses lower than 10 µM; these effects were abrogated by CB_2_ antagonism with SR-144,528 (Fig. 1b).

We next determined whether this discrepancy in dose response was associated with differences in baseline expression of genes in the endocannabinoid system between MDMs and iMg. The bulk RNA-seq analysis of gene expression based on counts normalized to per million reads, revealed that neither MDMs nor iMg expressed detectable levels of *CNR1*, the gene encoding CB_1_, on DPI 9 (Fig. 1c and d). Intriguingly, MDMs did not express *CNR2*, which was robustly expressed in iMg. *GPR55*, which encodes a putative cannabinoid receptor, was not expressed in iMg and was expressed in only one MDM donor (Masocha & Thomas, 2019; Morales & Reggio, 2017; Saliba et al., 2018). We also confirmed the RNA-Seq-based endocannabinoid receptor expression profile of MDMs with qRT-PCR (Supplementary Fig S3a).

Despite the undetectable *CNR2* expression, MDMs expressed very high levels of *MGLL*. This gene encodes monoacylglycerol lipase (MAGL, Fig. 1c and d), an enzyme primarily responsible for the hydrolysis of the endogenous CB_2_ ligand 2-arachidonoylglycerol (Grabner et al., 2016; Rojo-Bustamante et al., 2020; Zhang & Thayer, 2018). Conversely, the expression levels of *NAPEPLD* and *DAGLA*, which encode enzymes involved in endocannabinoid synthesis, and *FAAH*, the primary anandamide-hydrolyzing enzyme, did not differ between MDMs and iMg; the expression levels of these endocannabinoid system genes were unchanged by HIV infection or JWH-133 treatment (Fig. 1c and d). Given the observed difference in cannabinoid responsiveness between MDMs and iMg, we used 1 µM JWH-133, the only dose effective at impacting HIV infection in both cell types, for the subsequent experiments.

### CB_2_ Activation Reduces the Number of Infected Cells Without Altering Receptor and Coreceptor Expression Necessary for HIV Entry

HIV_ADA_ relies on CD4 and CCR5 for cellular entry (Michael et al., 1998; Trouplin et al., 2001). We determined whether the alteration in the expression of these receptors observed following JWH-133 exposure might contribute to the observed interference with HIV replication. CB_2_ agonism did not alter *CD4* or *CCR5* mRNA levels in MDMs as measured by RNA-seq, although HIV infection did downregulate *CD4* (Fig. 2a). This is a known phenomenon presumed to prevent superinfection (Benson et al. 1993; Lundquist et al., 2002). In iMg, *CD4* and *CCR5* mRNA levels were unchanged in any of the conditions (Fig. 2b). *CXCR4*, which encodes an HIV coreceptor not used by HIV_ADA_, was expressed at low levels in both cell types (Fig. 2a and b). Fig. S3 shows the qRT-PCR validation of the RNA-seq results in MDMs. By immunofluorescence staining, the relative fluorescence intensities of CD4 and CCR5 were not significantly different between JWH-133-treated/HIV-infected iMg and HIV-infected iMg at DPI 9 (Fig. 2c and d).

Reduced supernatant p24 l Gag levels could reflect a change in the production of virions from individual infected cells without a reduction in the total number of infected cells. Therefore, we used immunofluorescence staining with an antibody to HIV p24 Gag to determine the percentage of infected cells, defined as p24 Gag positivity, as well as the p24 Gag signal intensity/cell in iMg cultures. Reflecting the supernatant p24 Gag levels, JWH-133 significantly reduced the percentage of p24 Gag^+^ cells compared to cultures with HIV alone or to those co-administered SR-144,528 (Fig. 2e and f). These data suggested that CB_2_ activation reduced not only HIV p24 Gag production and release from cells but also the number of infected cells.

### MDMs are More Responsive than iMg To CB_2_-Mediated Immunoregulation

**Fig. 3:**
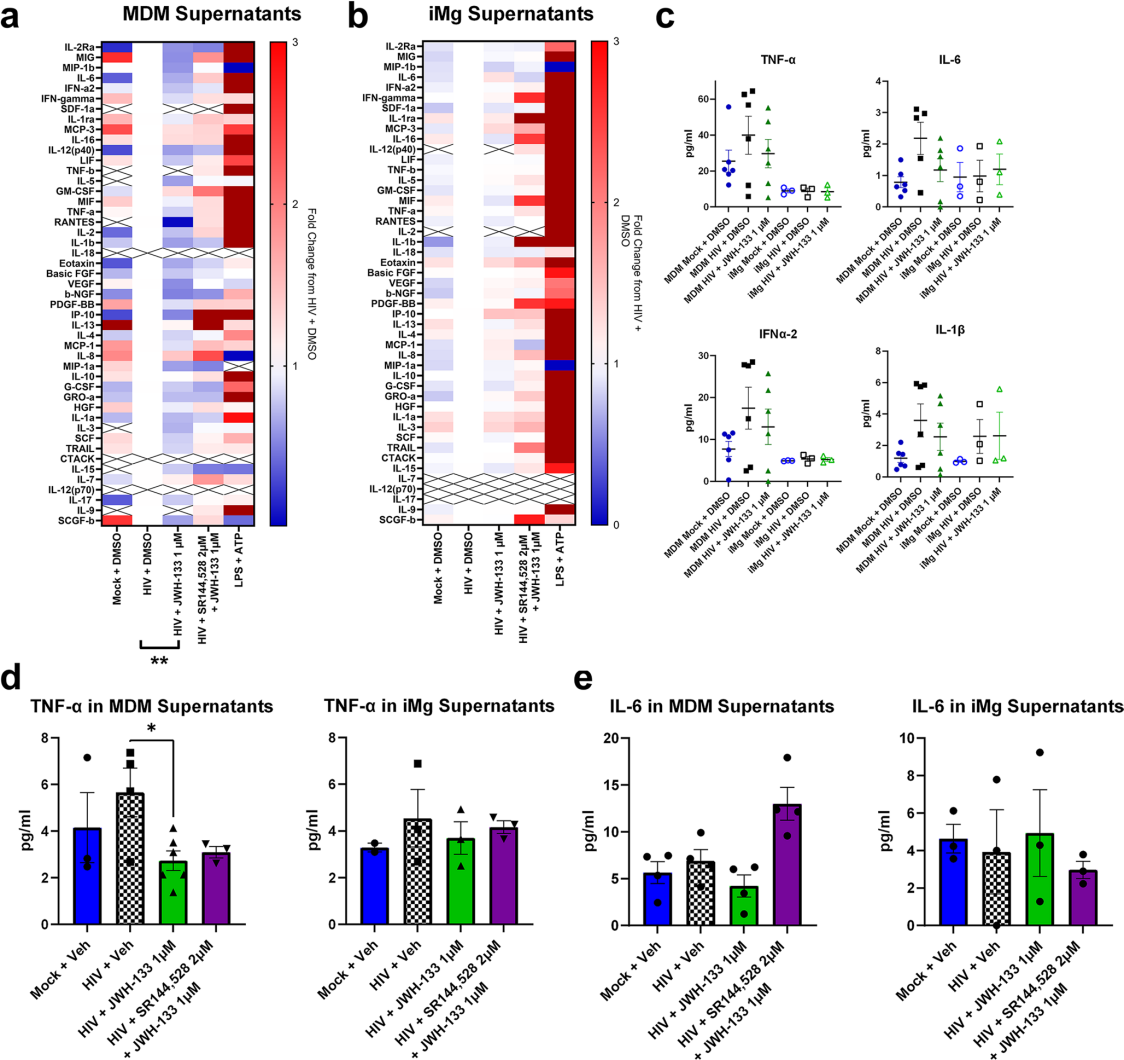
CB_2_ agonists reduce global cytokine release following HIV infection in MDMs but not in iMg.** a**,** b.** Cytokine levels in cell-free supernatants of MDM (**a**) and (**b**) iMg cultures were measured with Bio-Plex Pro 48-Plex human cytokine screening panel. Heatmaps show log_2_ fold changes from the HIV + vehicle condition. Supernatants were pooled from 4 wells/condition at days postinfection 9 (DPI 9) and assayed in duplicate. *n* = 6 MDM donors and 3 iMg lines. Student’s *t* test, **p < 0.01. **c.** Levels of cytokines reportedly associated with HIV-induced brain injury in MDM and iMg supernatants, measured with Bio-Plex Pro 48-Plex human cytokine screening panel. **d**,** e.** Levels of tumor necrosis factor alpha (TNF-α) and IL-6 in (**d**) MDM and (**e**) iMg culture supernatants, measured with AlphaLISA. *n* = 6 MDM donors and 3 iMg lines. Student’s *t* test, *p < 0.05

Considering the canonical function of CB_2_ as an immunoregulator, we determined whether JWH-133 reduced HIV-induced cytokine secretion in MDMs and iMg. While some studies have reported the downregulation of specific cytokines at both mRNA and protein levels, in this context, we initiated our analysis using an unbiased approach with a 48-plex enzyme-linked immunosorbent assay against a broad range of human cytokines and chemokines (Ehrhart et al., 2005; Persidsky et al., 2015). Because secreted cytokines are those most able to propagate inflammatory signals to neurons, other glia, and even other brain regions, we measured their concentrations in MDM and iMg culture supernatants (Fig. 3a and b). We found that 1 µM JWH-133 led to a global overall reduction in cytokine secretion from HIV-infected MDMs compared to the MDMs infected with HIV alone, an effect not observed in iMg (Fig. 3a and b). Globally, cytokine concentrations were higher in MDM cultures than in iMg cultures, even in mock-infected cells. This, combined with the lower HIV replication in iMg, might explain our finding that HIV did not robustly increase the levels of the same cytokines, such as TNF-α, IL-6, and interferon α2, in iMg as it did in MDMs (Fig. 3c). IL-1β, however, was increased in both MDMs and iMg infected with HIV. Due to this discrepancy in the inflammatory response to HIV, we were unable to determine whether the observed minimal iMg response to CB_2_ agonism was due to inherent cannabinoid biology in microglia, small maximal effect size, or potential differences in IL-1β regulation and secretion between primary microglia and iMg. Intriguingly, both MDMs and iMg displayed robust global response to inflammatory activation by LPS + ATP, indicating that muted microglial activation was specific to the HIV challenge (Fig. 3a and b).

To validate these unbiased observations, we measured the levels of IL-6 and TNF-α, which were previously reported to be altered by cannabinoid signaling (Costiniuk and Jenabian 2019; Nagre et al., 2022; Rizzo et al., 2020; Tanaka et al., 2020), in the same supernatant samples using AlphaLISA. These targeted assays reflected the general trends observed in the multiplex assay, such as a significant reduction in TNF-α levels released in MDM, but not iMg, supernatants following JWH-133 treatment; however, the quantitative values measured were not consistent between the AlphaLISA and multiplex assay (Fig. 3c-e). We considered the values measured using the targeted assays to be more convincing, since these assays had a wider standard curve range and were specific for one cytokine. It is also possible that differences in detection are attributable to differences in capture/detection antibodies between the assays, which are proprietary. Thus, while the unbiased assays proved useful for the assessment of broad trends, especially for our comparative analysis of MDMs and iMg, targeted assays were more useful for the investigation of specific inflammatory mediators.

### RNA-seq Reveals Divergent Responses To CB_2_ Agonism between MDMs and iMg

**Fig. 4:**
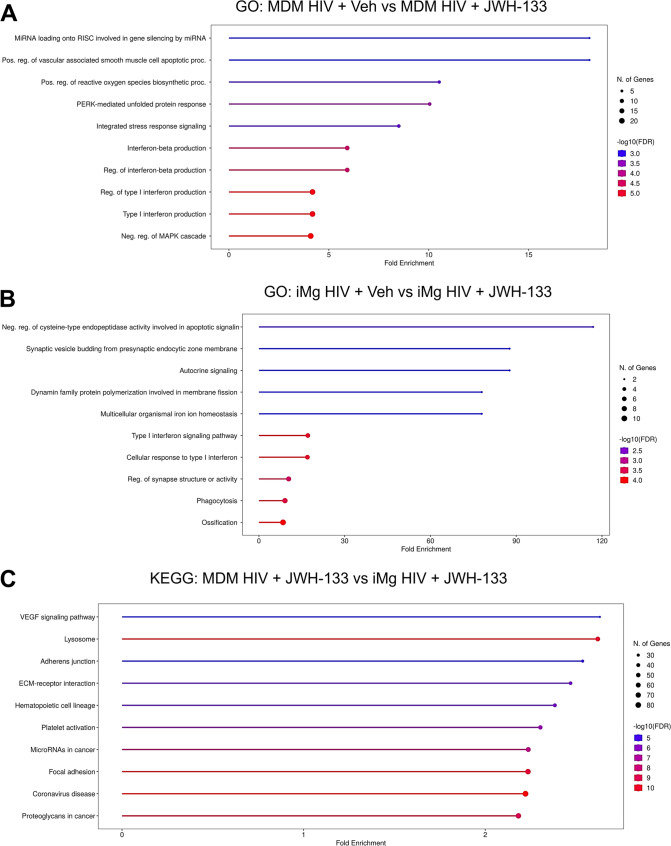
RNA-seq identifies convergent and divergent responses to CB_2_ signaling between HIV-infected MDMs and iMg.** a**,** b**. Top 10 GO biological process gene sets enriched in the significant differentially expressed genes (DEGs) in comparison between HIV-infected/vehicle-treated and HIV-infected/JWH-133 treated (**a**) MDMs and (**b**) iMg. **c.** Top 10 KEGG gene sets enriched in the significant DEGs in comparison between HIV-infected/JWH-133-treated MDMs and iMg. *n* = 2 MDM donors and 2 iMg lines

Although the immunoregulatory effects of CB_2_ signaling in HIV-infected myeloid lineage cells have been previously reported (Ramirez et al., 2013; Rock et al., 2007), specific biologic processes impacted by CB_2_ signaling, either directly or indirectly via immunoregulation, as well as the pathways uniquely altered by CB_2_ signaling in macrophages and microglia remain unclear. We employed bulk RNA-seq of DPI 9 MDMs and iMg that were mock-or HIV-infected with or without treatment with JWH-133 to identify significant DEGs and associated biologic pathways. Comparison of the HIV-infected MDMs to the HIV-infected MDMs treated with JWH-133 revealed that the most significantly altered GO: Biological Process pathways were interferon viral response, including *IRF5*, *STAT6*, and *IRAK1*, and integrated stress response (ISR), particularly the PERK-mediated unfolded protein response arm indicated by the significant upregulation of *ATF4*, *DDIT3*, and *EIF2AK3* (Fig. 4a and Supplementary Fig. S4a). While the former was expected based on our analyses showing decreased viral replication in JWH-133-treated HIV-MDMs, the latter observation was unexpected.

In HIV-infected MDMs and iMg treated with JWH-133, interferon response pathways were differentially expressed. Specifically, interferon response genes including *OAS3*, *IFIT1*, *IFIT2*, *MX1*, and *MX2*, were significantly downregulated in iMg, in contrast to the upregulation of the interferon response pathway observed in MDMs (Fig. 4b and Supplementary Fig. S4b). Because JWH-133 significantly reduced what was already a lower level of HIV replication in iMg than in MDMs, this finding might reflect the transcriptional resolution of antiviral response. Conversely, genes implicated in cell migration and phagocytosis, including *CCL2*, *CD93*, and *SLC11A1*, were upregulated (Fig. 4b and Supplementary Fig. S4b).

Finally, we compared the JWH-133-treated, HIV-infected MDMs to the JWH-133-treated, HIV-infected iMg to identify pathways that differed between the two cell types. For this cross-cell type analysis, we chose to examine both the GO: Biological Process and KEGG pathways, which include manually mapped pathways related to pathologic states. The majority of the top 10 GO terms were related to extracellular matrix interactions and cell motility, which also comprised three of the top ten KEGG pathways and was consistent with the upregulation of phagocytic pathways identified in the inter-microglial analysis (Fig. 4b and c and Supplementary Fig. S4c).

Intriguingly, we found considerable overlap between the GO-identified purinergic nucleotide receptor signaling pathway and the KEGG-identified coronavirus disease pathway, both of which were driven by the higher microglial expression of genes associated with the NLRP3 inflammasome. Considering ample evidence of NLRP3 inflammasome activation downstream of HIV infection in microglia (Chivero et al. 2017; He et al., 2020; Katuri et al., 2019; Mamik et al., 2017; Rawat et al., 2019; Thangaraj et al., 2018), studies showing that countering NLRP3 signaling is neuroprotective in HIV models (Jiang et al., 2022; Rawat et al., 2019), and emerging reports of interactions between cannabinoid signaling and the NLRP3 inflammasome (Gao et al., 2018; Jiang et al., 2022; Ke et al., 2016; Li et al. 2023; Nagre et al., 2022; Shao et al., 2014; Yu et al., 2019; Zhang et al., 2021), we further investigated their relationship in HIV-infected iMg.

### CB_2_ Suppresses NLRP3 Inflammasome Activation, But Not Priming, in HIV-Infected iMg

Canonically, NLRP3 inflammasome function occurs in two steps. In the first step, also termed priming, pathogen-associated molecular patters or cytokines mediate signaling through cell surface receptors, leading to the nuclear translocation of NF-κB and the subsequent transcriptional priming of genes that encode inflammasome components, including *NLRP3*, the scaffolding protein ASC (*PYCARD*), caspase 1 (*CASP1*), *IL1B*, *IL18*, and the pore-forming protein gasdermin D (*GSDMD*) (Albalawi et al., 2017; Bauernfeind et al., 2009; Coll et al. 2015; Swanson et al., 2019). Once inflammasome components are expressed, a second hit, often occurring via purinergic receptor signaling or mitochondrial stress, leads to the assembly of NLRP3 oligomers and ASC into the inflammasome, which cleaves caspase 1 and leads to the posttranslational processing of IL-1β, IL-18, and gasdermin D into their functional forms. Therefore, we first evaluated the nuclear translocation of NF-κB in response to HIV and JWH-133. While HIV infection led to an increase in nuclear translocation of NF-κB compared to the uninfected iMg, confirming the known impact of HIV on inflammasome priming (Chivero et al. 2017; Mamik et al., 2017; Walsh et al., 2014), JWH-133 did not alter the NF-κB nuclear translocation in HIV-infected iMg, although a certain extent of NF-κB nuclear translocation was also observed in uninfected iMg treated with JWH-133 (Supplementary Fig. S5).

**Fig. 5:**
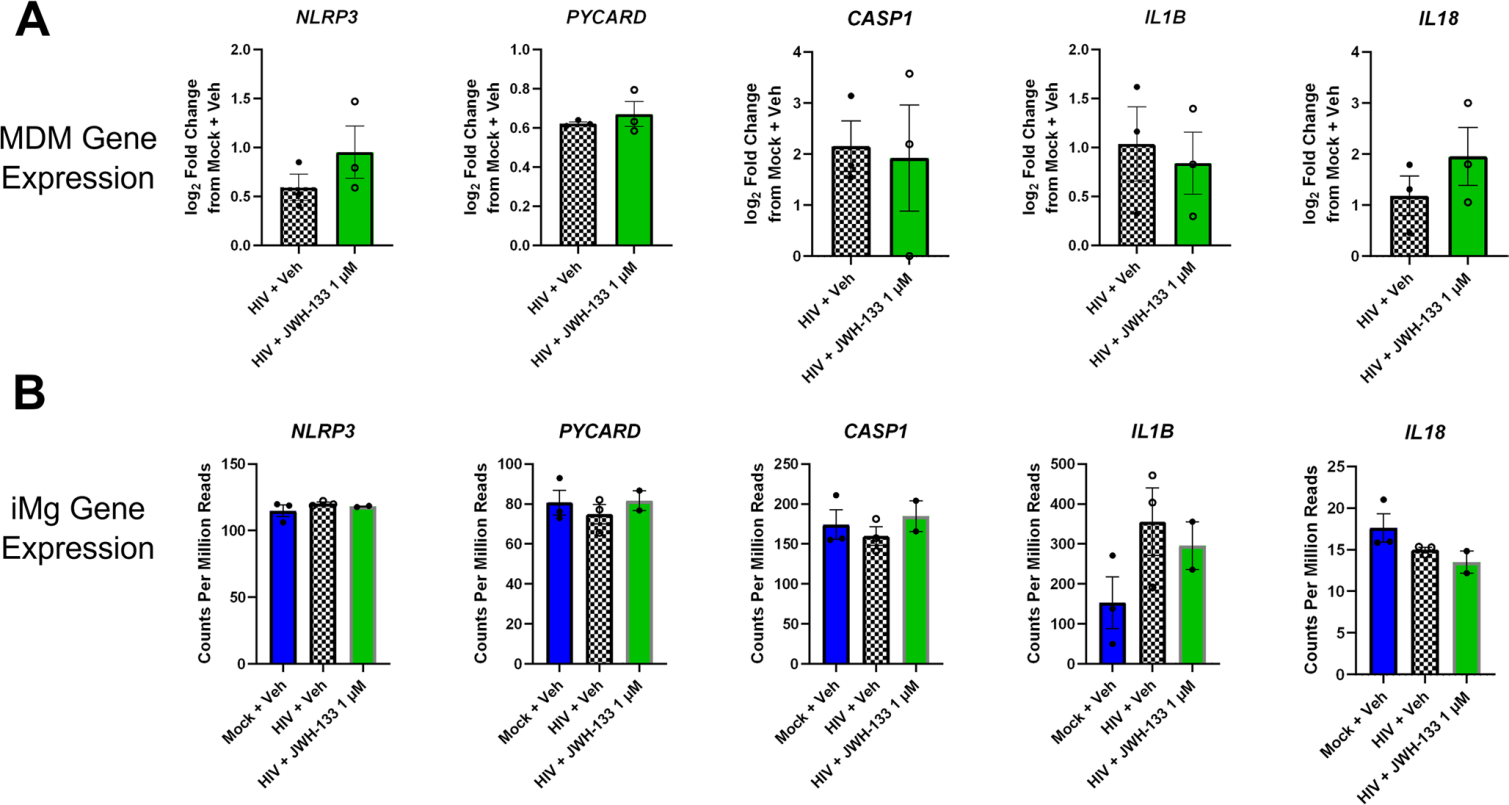
CB_2_ activation has minimal impact on transcriptional priming of the NLRP3 inflammasome in MDMs and iMg with established HIV infection. **(a)** NLRP3 inflammasome gene expression in MDMs on DPI 9, determined with qRT-PCR, represented as log_2_ fold change from Mock + Veh. *n* = 3 MDM donors. **(b)** NLRP3 inflammasome gene expression in iMg on DPI 9, determined with bulk RNA-seq. *n* = 3 iMg lines

**Fig. 6:**
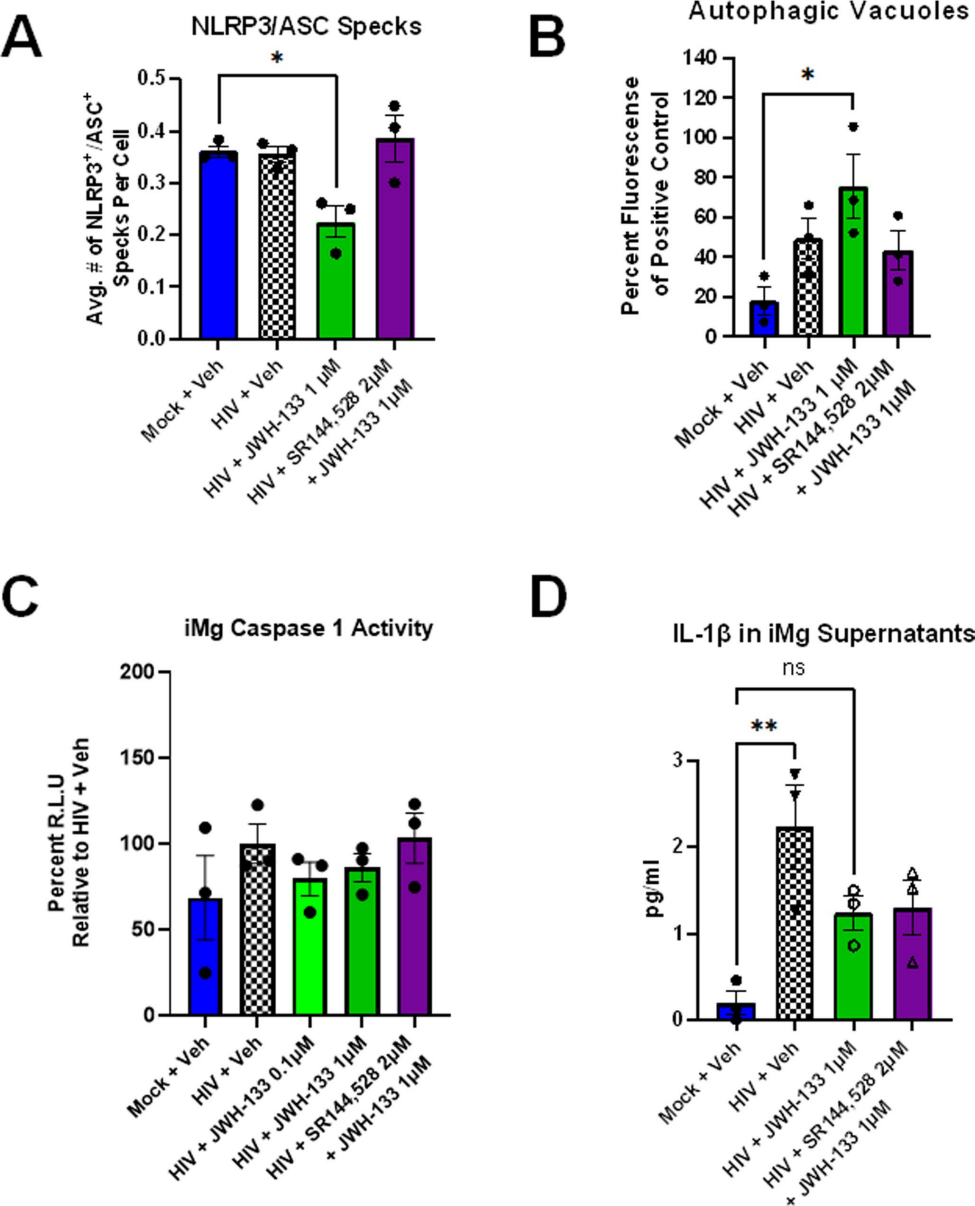
In iMg, CB_2_-specific agonists impair HIV-induced NLRP3 inflammasome activation with a corresponding increase in autophagosome number.** (a)** Quantification of the number of NLRP3^+^/ASC^+^ specks in iMg detected with immunostaining. Average of 2 wells/condition from 3 differentiations in 2 iMg lines, one-way ANOVA with Dunnett’s correction for multiple comparisons. * *p* < 0.05. **(b)** Autophagic vacuoles measured by an autophagy assay. *n* = 3 iMg lines, one-way ANOVA with Dunnett’s correction for multiple comparisons, *p < 0.05. **(c)** Caspase 1 activity measured by the Caspase-Glo 1 inflammasome assay, with results normalized to HIV + Veh. *n* = 3 iMg lines, one-way ANOVA with Dunnett’s correction for multiple comparisons, *p < 0.05. **(d)** Levels of IL-1β in iMg supernatants, measured by AlphaLISA. *n* = 3 iMg lines, one-way ANOVA with Dunnett’s correction for multiple comparisons, **p < 0.01; ns, not significant

Next, we examined the gene expression of the NLRP3 inflammasome components in both MDMs and iMg. At baseline, both iMg and MDMs expressed *NLRP3*, *PYCARD*, *CASP1*, and *IL1B* (Fig. 5a and b). Given that NLRP3 inflammasome activation can be decoupled from priming (Gritsenko et al., 2020; Juliana et al., 2012), we next determined the formation of NLRP3^+^/ASC^+^ specks, constituents and indicators of inflammasome formation and activation (Nagar et al., 2023), by immunostaining in HIV-infected iMg. At DPI 9, the number of NLRP3^+^/ASC^+^ specks/cell did not differ between the HIV- and mock-infected iMg. However, the addition of JWH-133 significantly reduced the number of NLRP3^+^/ASC^+^ specks/cell in HIV-infected iMg even below that observed in mock-infected and vehicle-treated iMg (Fig. 6a). The observed decrease in the number of NLRP3^+^/ASC^+^ specks was observed in parallel with JWH-133-induced increase in autophagy (Fig. 6b), consistent with studies reporting that autophagy downregulates NLRP3 inflammasome activation by phagocytosing inflammasome assemblies (Jiang et al., 2022; Rawat et al., 2019; Shao et al., 2014).

The ultimate consequence of NLRP3 inflammasome activation is caspase 1-mediated processing and release of IL-1β and IL-18, (which was not detected in MDM or iMg supernatants. As shown in Fig. 6c, the increase in caspase 1 activity following HIV infection was not significantly higher than that observed in controls, although a dose-dependent increase in caspase 1 activity was observed in JWH-133-treated cultures.

We also evaluated the secretion of IL-1β as another marker for NLRP3 inflammasome activation. While IL-1β secretion from iMg was not significantly reduced in our assay including a large number of cytokines (Fig. 3c), targeted measurement using AlphaLISA revealed that HIV infection significantly increased IL-1β release compared to the mock controls and that JWH-133 treatment reversed IL-1β secretion by approximately 50% to below the level of significance (Fig. 6d).

Altogether, these data suggested that dose-dependent CB_2_ signaling was a negative regulator of inflammasome activation, but not priming, in HIV-infected iMg.

## Discussion

Combating CNS HIV reservoirs requires therapies which can cross the blood-brain barrier and address both viral replication and downstream cytotoxic pathways. While the therapeutic use of phytocannabinoids has been increasing in popularity across a wide range of diseases, the activity of Δ9-THC and CBD on CB_1_ may not be ideal for neurocognitive disorders where neurotransmitters are already dysregulated (Costiniuk et al. 2019; Tyree et al., 2019; Whiting et al., 2015; Wilkinson et al., 2016). CB_2_-specific agonists avoid psychoactive CB_1_ activity while continuing to offer the immunomodulatory effects of cannabinoids. As this study indicates, CB_2_-specific agonists are also capable of reducing both HIV infection and multiple inflammatory pathways implicated in HIV-associated neuronal damage and dysfunction.

Several studies have shown that cannabinoid signaling can interfere with HIV infection in macrophages and microglia via different mechanisms (Fraga et al., 2011; Persidsky et al., 2015; Ramirez et al., 2013; Rock et al., 2007). Importantly, the models and pharmacologic agents in these studies frequently included potential dual-receptor activity, as both CB_1_ and CB_2_ and receptor-specific antagonists/inverse agonists were shown to exert specific effects on key infection readouts. Therefore, we compared HIV infection in MDMs and iMg that do not express CB_1_ transcripts, which were only exposed to CB_2_-specific ligands. To that end, we used JWH-133, a synthetic cannabinoid with a Ki of 3.4 nM and > 200-fold selectivity for CB_2_ over CB_1_, and SR-144,528, a CB_2_-selective antagonist/inverse agonist with a Ki of 0.6 nM and > 600-fold selectivity for CB_2_ over CB_1_. Importantly, both compounds exhibit minimal off-target effects at concentrations of up to 10 µM compared to other compounds used in previous studies (Soethoudt et al., 2017).

We did not detect *CNR2* expression in MDMs on DPI 9, which might be related to the time- and activation-dependent expression of *CNR2* in macrophages, as previously reported (Benito et al., 2005; Carlisle et al. 2002; Coopman et al. 2007; Cosenza-Nashat et al. 2011; Galan-Ganga et al., 2020; Grabon et al., 2023) CB_2_ pharmacologic activity has been reported in activated immune cells, in which *CNR2* mRNA is downregulated or not detected; one possibility is that functional receptor expression might be decoupled from active gene expression. Although determining CB_2_ receptor expression at the protein level would further strengthen our conclusions, our attempts to detect CB_2_ in MDMs and iMg by immunofluorescence staining using two antibodies most likely to detect CB_2_ receptor expression at the protein level in human cells (cat. no: 101550, Cayman Chemical (Grabon et al., 2023) and cat no: FAB36551R; R&D Systems (Rosario-Rodríguez et al., 2022) were unsuccessful and represent a major technical barrier widely recognized in the field and summarized in a recent review (Grabon et al., 2023).

The observed high *MGLL* expression levels in MDMs raise the possibility that MDMs may be transcriptionally primed to exhibit lower sensitivity to CB_2_ agonists than iMg, which express higher *CNR2* expression and lower levels of the enzyme that regulates endogenous CB_2_ agonism, consistent with our observation that the regulation of HIV replication in MDMs occurred at higher JWH-133 doses in comparison to iMg, which exhibited optimal response at lower JWH-133 doses.

We did not detect differences in HIV receptor/coreceptor expression levels between iMg and MDMs, consistent with studies suggesting that cannabinoid regulation of HIV infection does not include the viral entry step (Ramirez et al., 2013).

Upregulation of several genes involved in the ISR pathway observed in JWH-133-treated MDMs was not expected. Interactions between the ISR and CB_2_ signaling have not been well studied, and current evidence on the precise impact of CB_2_ signaling on the activation of stress response is conflicting (Almada et al., 2020; Li et al., 2022). Indeed, given the tight temporal regulation of ISR and its biphasic activity (Batjargal et al., 2023), CB_2_ might not specifically impact the ISR and the implications of ISR dysregulation in cellular outcomes may be indirect. Downregulation of interferon response pathways in JWH-133-treated iMg is consistent with our previous findings that HIV infection impairs phagocytic function in iMg, with CB_2_ acting as a negative regulator of HIV-associated inflammation (Ryan et al., 2020).

Higher baseline expression levels of inflammasome-related genes in iMg are likely related to the differences observed in the HIV + JWH-133 group by RNA-seq, as neither HIV infection nor cotreatment with JWH-133 significantly altered the expression levels of any of the inflammasome-related genes, confirmed by qRT-PCR (Fig. S3b). The lack of inhibition of NF-κB translocation in HIV-infected iMg by JWH-133 suggests that JWH-133 does not impact priming in our long-term infection/treatment paradigm.

In iMg, we observed an increase in caspase 1 activity at the highest JWH-133 dose, although it remains unclear whether this is a cause or effect of increased HIV replication. Notwithstanding, the attenuation of the IL-1β secretion by HIV-infected iMg following JWH-133 treatment suggests that dose-dependent CB_2_ signaling is a negative regulator of inflammasome activation, but not priming, in HIV-infected iMg.

This is the first in vitro study including the side-by-side comparison of the antiviral and immunomodulatory functions of CB_2_ in models of macrophages and microglia, two cell types considered important CNS HIV reservoirs. While their shared myeloid lineage suggests functional overlap, our results indicate their susceptibility to HIV infection, cytokine secretion, and transcriptomic responses differ as do their responses to CB_2_ signaling. Although general trends were shared between the two cell types, additional aspects, including the pharmacokinetics of CB_2_ agonists within the periphery and the CNS and their impact across different cell types should be considered in future in vivo studies exploring the therapeutic utility of CB_2_ agonism.

The majority of CB_2_-expressing cells are within the periphery, and peripheral HIV reservoirs, including those in the gut, spleen, and lymphatic tissue, can benefit from the antiviral effects of CB_2_ agonism. However, there is less of a distinct need for CB_2_ agonists’ antiviral effect in the periphery, where antiretrovirals are more effectively distributed in tissues and are very successful in controlling viral replication. Moreover, although the anti-inflammatory effects of CB_2_ agonists may be desirable to mitigate inflammation in the periphery, these must be balanced against their potential adverse impact on immune responses necessary to combat pathogens. Considering that the periphery encounters far more microbes than the CNS, stunting the peripheral immune response, exemplified by the global reduction in cytokine release we observed in JWH-133-exposed HIV-infected MDMs, with CB_2_ agonism requires a careful approach especially in people with HIV who are at higher risk for opportunistic infections. Thus, treatment paradigms that leverage cannabinoid lipophilicity to achieve effective concentrations in the brain while maintaining low peripheral concentrations and those that utilize targeted therapeutic delivery to the CNS, such as nanoparticles targeted to specific cell types, may be preferable.

CNS-targeted approaches should consider the differences in the baseline endocannabinoid system expression and sensitivities to the CB_2_ agonist doses we observed between MDMs and iMg. Postmortem studies of the relative contribution of each cell type to the CNS viral load are not well established, with many studies using pan-macrophage markers, such as CD68, that prevent any delineation. However, considering HIV- infected myeloid cells are found both in the perivascular space and the parenchyma, it is likely that both macrophages and microglia contribute to the neuropathology of HIV. Thus, lower doses of CB_2_-specific agonists that do not overwhelm microglia are preferable to achieve some beneficial effect in perivascular macrophages, which may in fact “see” a higher effective concentration than parenchymal microglia, due to macrophages’ proximity to the blood stream. Of course, properly designed in vivo studies are necessary to better inform both the relative contribution of these cell types and the pharmacokinetics of CB_2_ agonists in brain-resident myeloid cells.

Since our work assessed the impact of CB_2_ agonism on HIV-relevant cell types individually to elucidate the cell type-intrinsic effects, future studies should compare the impact of CB_2_ agonism between the individual cell types and the cocultures of the two cell types. While ART will likely remain the primary antiviral treatment for HIV, our work suggests that CB_2_ agonists may provide a boost to their antiviral function. Another key consideration that should be prioritized in studies exploring the potential combination of CB_2_ agonists with ART is their impact on CYP450 and other drug-processing enzymes, which may impact their pharmacokinetics and interactions with other drugs.

While we were careful to choose receptor-specific pharmacologic agents with minimal off-target effects, reproducing these studies across a range of CB_2_ agonists would enhance our understanding of dosing and potential off-target effects. In addition, while our findings have relevance to the CB_2_-signaling component of cannabis use, the complex actions of CBD as a negative allosteric modulator acting in the presence of more than 80 other phytocannabinoids found in cannabis limits the translatability of our results in the context of cannabis use or abuse (Aly et al., 2019; Navarro et al., 2018; Stout & Cimino, 2014).

HIV strain and inoculum should also be considered in the generalizability of our results. HIV strains vary in their cell-type tropism and infection efficiency, which also impact downstream molecular responses to infection. For example, in a previous study, we used HIV_Jago_, rather than HIV_ADA_, at 50X higher inoculum, for iMg infection, resulting in p24 Gag immunopositivity in nearly 95% of the cells, higher than the maximum of 50% observed in the present study (Ryan et al., 2020). Additionally, HIV_Jago_ infection significantly upregulated TNF-α in iMg in our previous study whereas TNF-α was not significantly upregulated in unbiased and targeted assays performed in iMg infected with HIV_ADA_. These observations highlight the context-dependent nature of immune responses and the value of repeating similar studies in different paradigms to identify robust, generalizable molecular targets.

Given that microglia are not usually exposed to serum in physiologic context, it remains possible that the activation of iMg in response to exposure to serum in culture medium might have contributed to our observations. While our model has been built on our earlier work demonstrating that the iMg used herein closely recapitulate freshly isolated human microglia (Ryan et al., 2020), future work should elucidate the impact of CB2 agonism on HIV replication by comparing iMg maintained in serum-containing conditions to those maintained in serum-free conditions.

This study is the first, to our knowledge, to demonstrate that a CB_2_-specific agonist regulates the NLRP3 inflammasome in HIV-infected iMg; only one study demonstrated this effect in LPS-treated microglial cell lines (Jiang et al., 2022). In addition, CBD was recently shown to downregulate the NLRP3 inflammasome in HC69.5, a microglial cell line with integrated HIV reporter virus (Yndart Arias et al., 2023). One advantage of iPSC-based models is that the findings can be expanded to isogenic multicellular models, including cocultures and organoids, to study the impact of CB_2_ microglial immunoregulation on nearby glia and neurons (Dolmetsch & Geschwind, 2011; Engle et al., 2018; Qian et al., 2016; Ryan et al., 2020; Sabitha et al., 2021). iPSC macrophage models are also well established and can be used alongside isogenic iMg models to parse out myeloid cell type-specific responses in more depth (Bernareggi et al. 2019; Gutbier et al., 2020; Lee et al., 2018).

These findings also have implications for neuroinflammatory conditions outside of HAND. Limited reports have studied NLRP3 regulation by CB_2_ in autoimmune encephalomyelitis, spinal cord injury, and alcohol-associated anxiety (Jiang et al., 2022; Li et al. 2023; Shao et al., 2014), but microglia-mediated NLRP3 inflammasome activation has been implicated in a number of other neurologic disorders, including Alzheimer’s disease, Parkinson’s disease, amyotrophic lateral sclerosis, traumatic brain injury, and stroke as well (Feng et al., 2020; Guan & Han, 2020; Lu et al., 2022; O’Brien et al., 2020; Piancone et al., 2021; Voet et al., 2019). Should the observations reported here prove generalizable to other neuroinflammatory conditions, CB_2_-specific agonists may be developed as useful therapeutics across acute and chronic neurologic disorders.

## Supplementary Information

Below is the link to the electronic supplementary material.

Supplementary Material 1 (PDF 801 KB)

## Data Availability

All sequencing data are available in the NCBI GEO database (https://www.ncbi.nlm.nih.gov/geo/) with the accession number GSE250616. Other data used and/or analyzed during the current study are available from the corresponding author on reasonable request.
