# Supplementary material for: Differential Effects of Cannabinoid Receptor 2 Agonists on HIV Replication and Inflammatory Activation in Monocyte-Derived Macrophages and Induced Pluripotent Stem Cell-Derived Microglia
Source: J Neuroimmune Pharmacol. 2025 Oct 11;20(1):87. doi: 10.1007/s11481-025-10254-x (PMC12515222; doi:10.1007/s11481-025-10254-x)
Supplement: Supplementary file 1 — Supplementary Material 1 (PDF 801 KB) [file 11481_2025_10254_MOESM1_ESM.pdf]

Differential effects of cannabinoid receptor 2 agonists on HIV replication and inflammatory activation in monocyte-derived macrophages and induced pluripotent stem cell-derived microglia

Alexander Starr[1] (ORCID: 0000-0002-0756-4082), Sara Rathore[1], Marzieh Daniali[2] (ORCID: 0000-0001-7703-8777), Peter J. Gaskill[2] (ORCID: 0000-0003-0095-5424), Cagla Akay-Espinoza[1] (ORCID: 0000-0003-0199-3836), Kelly L. Jordan-Sciutto[1] (ORCID: 0000-0002-0827-1337)

[1] Department of Oral Medicine, School of Dental Medicine, University of Pennsylvania, Philadelphia, PA 19104

[2] Department of Pharmacology & Physiology, Drexel University College of Medicine, Philadelphia, PA 19102

Corresponding author:

Kelly L. Jordan-Sciutto

Department of Oral Medicine, School of Dental Medicine, University of Pennsylvania

240 S. 40th St, Rm 312 Levy, Philadelphia, PA 19104

Phone: 215-898-4196; E-mail: jordank@upenn.edu

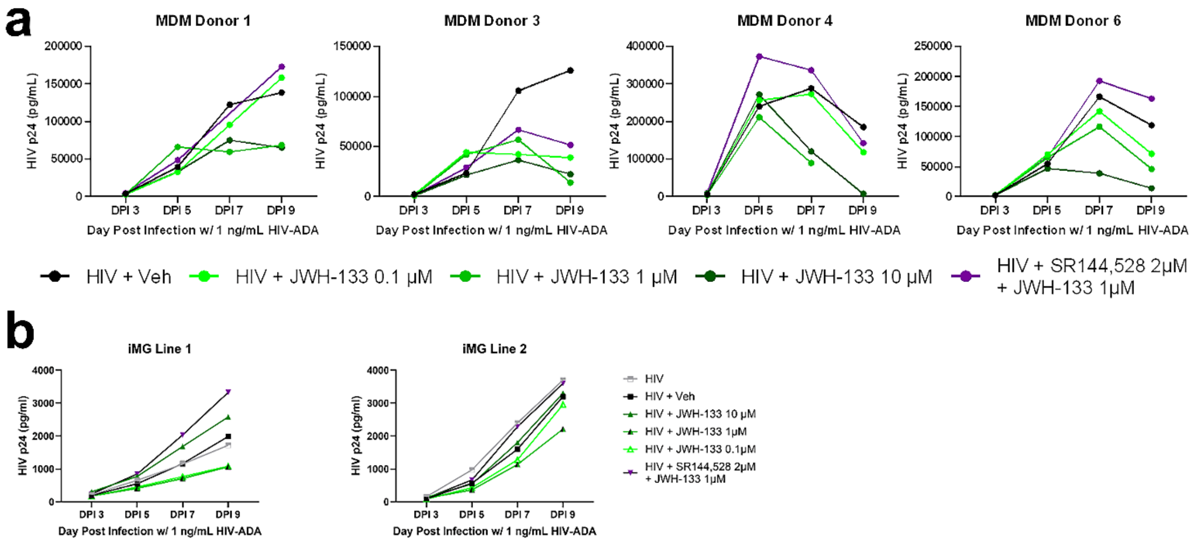

Supplementary Figure S1. CB_2 agonists dose-dependently reduce HIV replication in macrophages and microglia. Quantification of HIV p24 levels in supernatants of human monocyte-derived macrophages (MDMs) (a) and human induced pluripotent stem cell-derived microglia (iMg) (b), measured by AlphaLISA

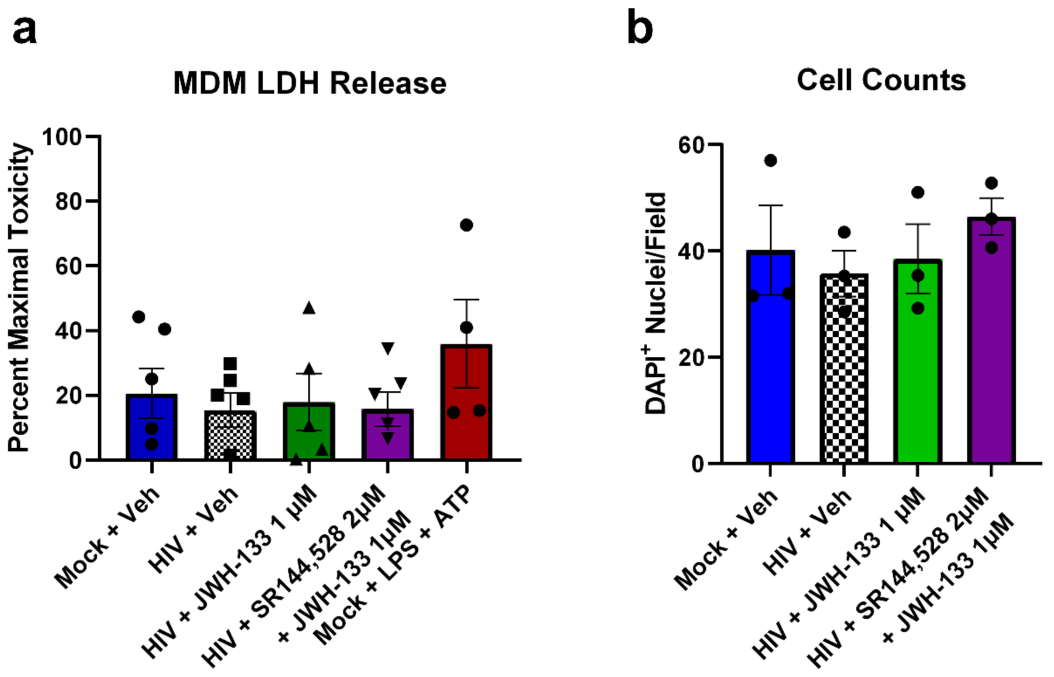

Supplementary Figure S2. HIV_{ADA} and CB_2-specific cannabinoids are not cytotoxic in MDMs and iMg at DPI 9. a. LDH release in MDM supernatants was quantified using the Cytotoxicity Detection kit. Data are presented as percentage of maximal cell toxicity in positive controls. n = 5 MDM donors. **b.** $DAPI^+$ iMg nuclei are averaged from 5 wells/condition across 3 differentiations.

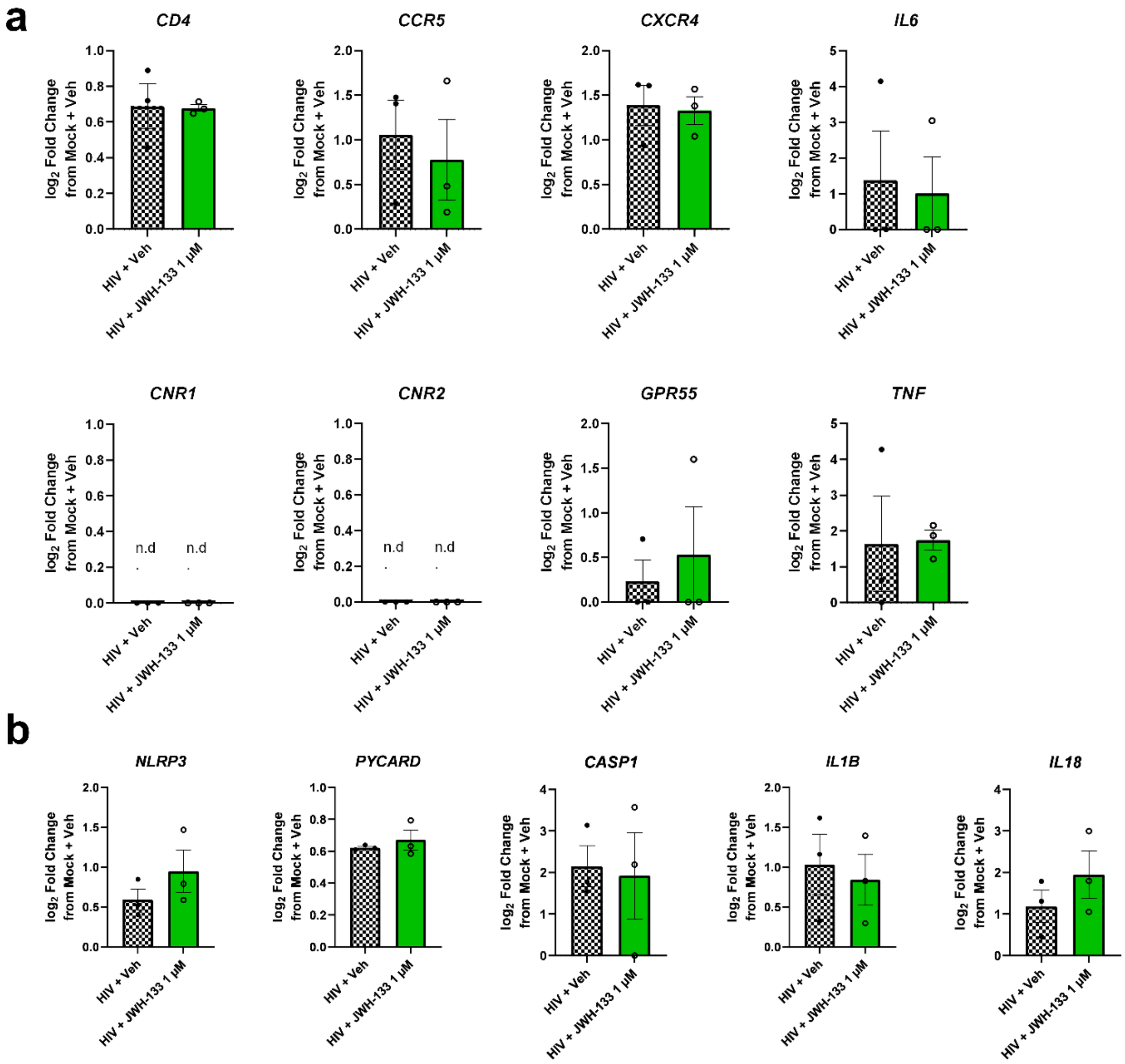

Supplementary Figure S3. RNA-seq transcript counts are validated by qRT-PCR. a. Expression of key HIV entry, endocannabinoid system, and cytokine transcripts in MDMs, measured by qRT-PCR, represented as fold change from Mock + Veh, n = 3 MDM donors. **b.** Expression of NLRP3 inflammasome transcripts in MDMs, measured by qRT-PCR, represented as fold change from Mock + Veh, n = 3 MDM donors

n.d., not detected

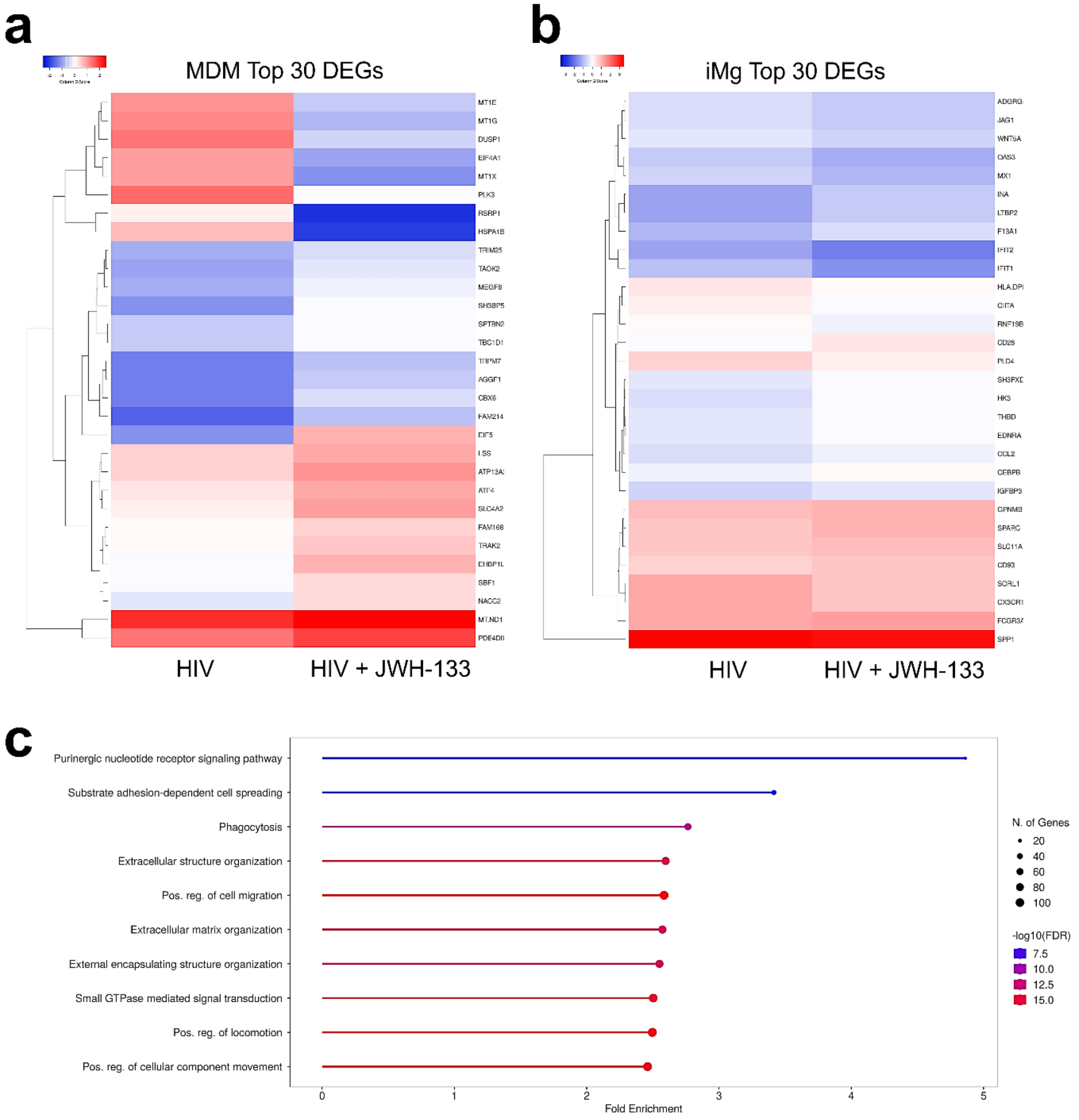

Supplementary Figure S4. RNA-seq identifies divergent responses to CB_2 signaling across HIV-infected myeloid cell cultures. a. Heatmap showing Manhattan plot clustering of log_2 normalized read counts per million of the top 30 significant differentially expressed genes (DEGs) in HIV + DMSO vs HIV + JWH-133 MDM, **b.** HIV + DMSO vs HIV + JWH-133 iMg. **c.** Top 10 GO Biological Process gene sets that are enriched in the

significant DEGs of HIV + JWH-133 MDM vs HIV + JWH-133 iMg. n = 2 MDM donors, n = 2 iMg lines

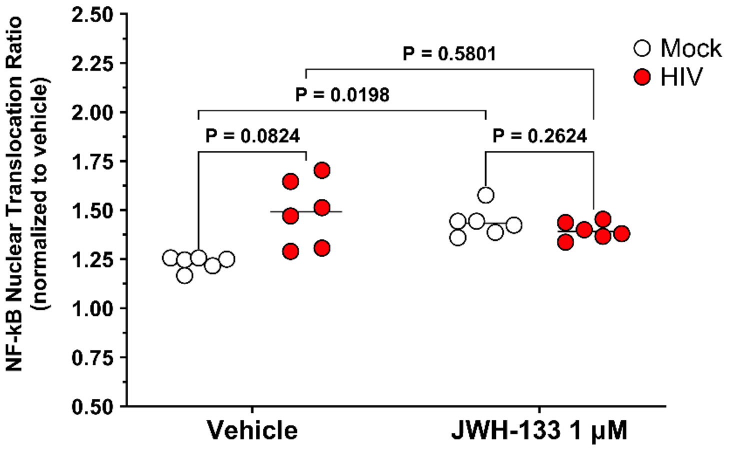

Supplementary Figure S5. CB_2-specific agonists do not suppress NLRP3 inflammasome priming in HIV-infected iMg. Data showing the ratio of iMg with NF-κB nuclear translocation determined using immunofluorescence-based NF-κB translocation assay. Average of 3 wells/conditions from 2 differentiations in 3 iMg lines, two-way ANOVA with Tukey's correction for multiple comparisons
